# Giant Exophytic Gastrointestinal Stromal Tumor (GIST) Arising From the Antral Stomach: A Surgical Case Report

**DOI:** 10.7759/cureus.48773

**Published:** 2023-11-14

**Authors:** Simran Chauhan, Raju K Shinde, Yashraj Jain

**Affiliations:** 1 General Surgery, Jawaharlal Nehru Medical College, Datta Meghe Institute of Higher Education and Research, Wardha, IND

**Keywords:** multidisciplinary approach, antral stomach, diagnostic imaging, surgical management, exophytic, gastrointestinal stromal tumor (gist)

## Abstract

This comprehensive case report meticulously outlines the clinical manifestation, diagnostic trajectory, surgical intervention, pathology, chemotherapy, and patient follow-up in a challenging instance of a giant exophytic gastrointestinal stromal tumor (GIST) emerging from the antral part of the stomach in a 56-year-old male patient. Over the course of a year, the patient presented with symptoms including abdominal distension, a progressively enlarging lump, constipation, and abdominal fullness. Our diagnostic strategy, anchored by ultrasonography and contrast-enhanced computed tomography (CECT), yielded crucial insights into the tumor's precise dimensions and anatomical location. The subsequent surgical planning and execution were pivotal, entailing a meticulous dissection of the cystic mass from adjacent structures to ensure negative margins. Histopathological scrutiny of frozen sections from the lymph nodes and excised antral margins conclusively confirmed the absence of malignancy, facilitating primary closure. This case report underscores the decisive role of accurate diagnostic imaging in shaping surgical strategies and emphasizes the need for a multidisciplinary approach to managing GISTs. Beyond the surgical realm, the case highlights the significance of tyrosine kinase inhibitors (TKIs), particularly imatinib, in the treatment paradigm. Additionally, the report sheds light on ongoing research endeavors to refine treatment modalities in GISTs.

## Introduction

Gastrointestinal stromal tumors (GISTs) are rare neoplasms originating from the gastrointestinal tract, with the stomach being one of the primary sites of occurrence [1]. Gastrointestinal stromal tumors have garnered significant attention in the medical community due to their unique clinical and pathological characteristics and advancements in diagnostic modalities and therapeutic approaches [2].

Characterized by their expression of the c-kit proto-oncogene protein (CD117), GISTs represent a subset of mesenchymal tumors originating from the gastrointestinal wall, often involving the stomach and small intestine [3]. These tumors are known for their variable clinical presentations and unpredictable malignant potential, ranging from indolent to highly aggressive. While some GISTs remain asymptomatic, others manifest as abdominal pain, bleeding, obstruction, or a palpable abdominal mass [4]. Gastrointestinal stromal tumors are rare, making up less than 1% of all gastrointestinal tumors. Approximately 4,000 to 6,000 adults in the United States will be diagnosed with a GIST each year. About 60% of GISTs begin in the stomach, and around 35% develop in the small intestine [5].

The management of GISTs has been revolutionized by the advent of tyrosine kinase inhibitors (TKIs) like imatinib mesylate (Gleevec), which target the molecular abnormalities driving GIST proliferation [6]. Although surgical resection remains the primary curative modality for localized GISTs, adjuvant imatinib therapy has proven to enhance disease-free survival [7] significantly.

This case report focuses on a patient with a notably large and exophytic GIST originating from the antral part of the stomach, emphasizing the importance of precise diagnosis and timely surgical intervention. A multidisciplinary approach, integrating diagnostic imaging, histopathological analysis, and surgical expertise, was pivotal in successfully managing this patient. The case underscores the critical role of accurate diagnosis, individualized treatment strategies, and the evolving landscape of GIST therapy.

## Case presentation

A 56-year-old male patient, devoid of known comorbidities, presented with a one-year history of complaints regarding an abdominal lump accompanied by abdominal distension. This lump exhibited progressive growth and was associated with symptoms of constipation and abdominal fullness. The patient demonstrated good tolerance for liquid and solid diets, with no discernible history of significant appetite loss or weight reduction.

The patient's abdomen was distended upon clinical examination, featuring a conspicuous protrusion in the right hypochondrium and epigastric region. The umbilicus appeared inverted, with no apparent signs of dilated veins or significant cutaneous alterations. Moreover, no inguinoscrotal swellings were detected. Upon palpation, the abdominal lump was identified as a single, sizable, non-tender mass, displaying a solid-cystic consistency and mobility synchronized with respiration.

Ultrasonography (USG) of the abdomen and pelvis revealed a large, solid cystic lesion measuring approximately 30 x 15 x 14 cm, affecting all four quadrants, including the hypochondrium and left iliac fossa. Doppler imaging unveiled areas of vascularity within the lesion, which was exerting a mass effect on the liver, compressing it superiorly and posteriorly.

**Figure 1:**
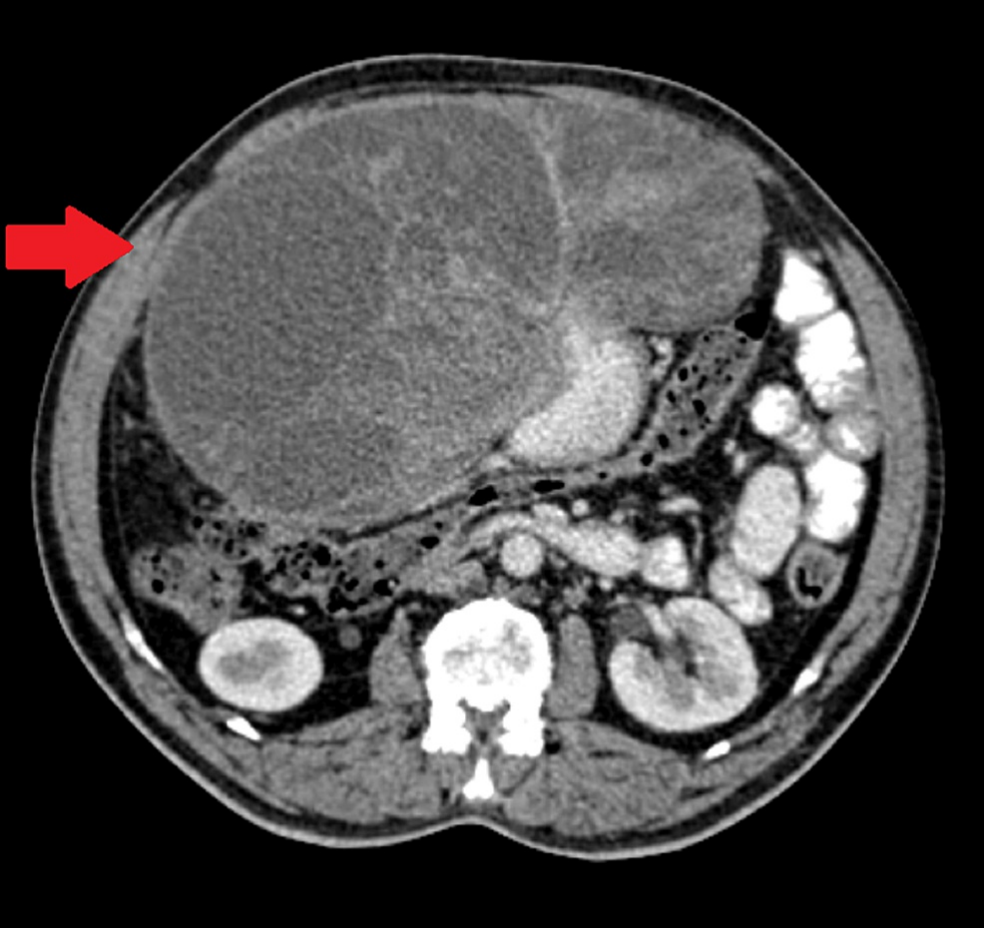
Swelling seen with a solid cystic component with lobulations arising from the antral wall

Following the initial imaging evaluation, a comprehensive contrast-enhanced computed tomography (CECT) scan of the abdomen and pelvis was undertaken. The CECT findings revealed a noteworthy and intricate depiction of the tumor's characteristics. Notably, the mass appeared large, lobulated, and relatively well-defined, displaying heterogeneous features suggestive of both cystic alterations and internal septations (Figure 1).

This presented a unique aspect, raising questions about the coexistence of cystic components in conjunction with a neoplastic process, especially considering the tumor's origin in the gastric mucosa. Further complexity was introduced by identifying hyperdense areas within the mass, indicative of potential hemorrhages. Post-contrast imaging delineated peripheral enhancement, extending not only to the cystic elements but also to the solid internal components of the mass. Additionally, the radiological examination revealed the presence of several sub-centimetric mesenteric lymph nodes, which were cautiously interpreted as likely representing a reactive state rather than manifesting malignant involvement.

**Figure 2:**
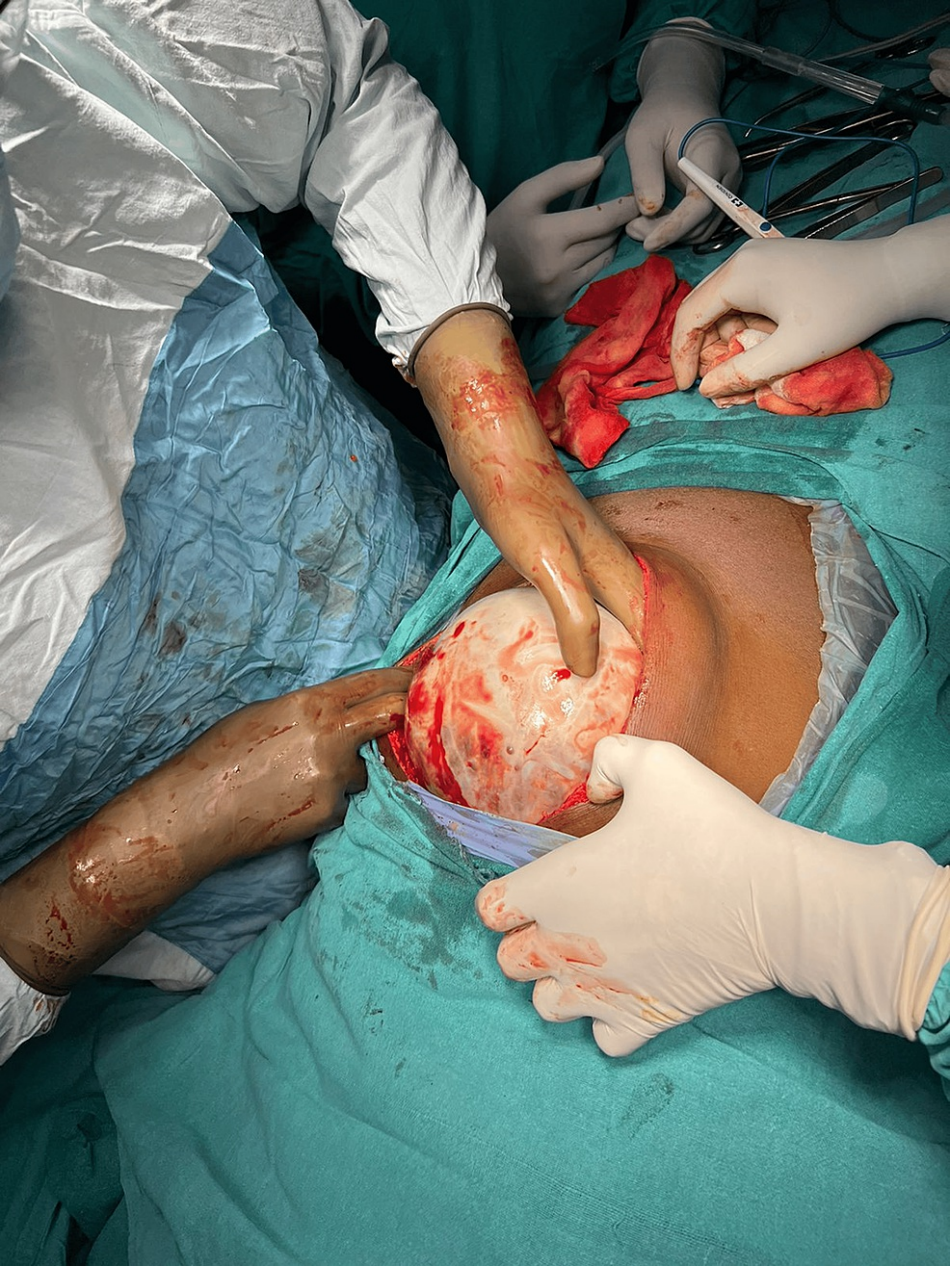
The mass is seen with a solid-cystic peritoneal wall

**Figure 3:**
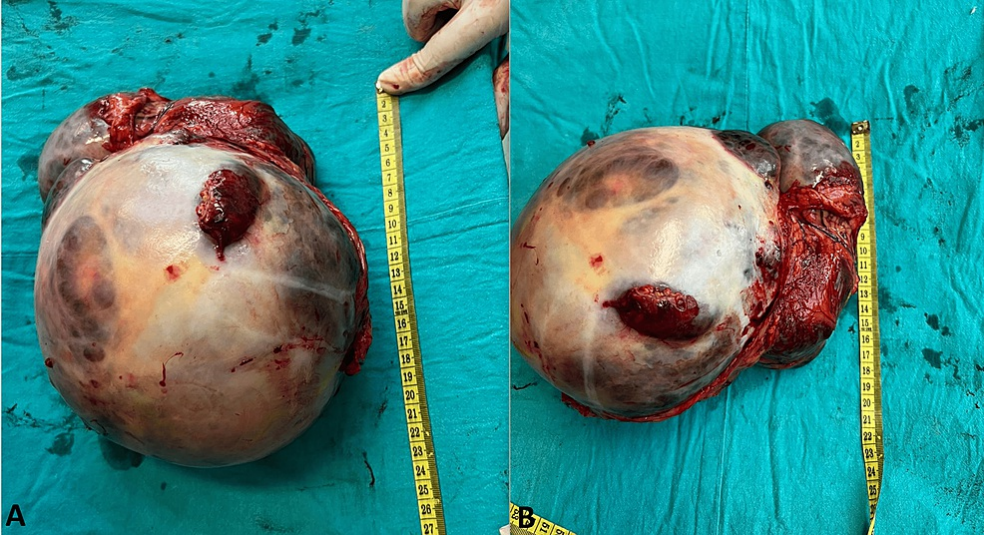
Panels A and B show the substantial component, measuring 20 x 15 x 16 cm.

Considering the diagnostic findings, the patient was scheduled for a laparotomy to excise the abdominal lump originating from the stomach. During the surgical procedure (Figure 2), a formidable mass with solid-cystic consistency, measuring 25 x 15 cm, was identified (Figure 3).

This bilobed mass was found to originate from the antral part of the stomach and was meticulously separated from the mesentery and the antral portion of the stomach. Subsequently, margins from the antral wall were excised and submitted for a frozen section for histopathological evaluation.

The histopathological examination of the frozen sections, an integral part of the intraoperative interventions in this case, provided critical insights into the nature of the specimens. Evaluation of the lymph node frozen section revealed characteristics consistent with reactive lymphadenitis, reassuringly devoid of any signs indicative of malignancy. Similarly, the frozen section analysis of the excised antral margins of the stomach yielded no evidence of malignant cell infiltration. Armed with these real-time diagnostic assessments, we proceeded confidently to execute the primary closure of the antral wall.

Postoperatively, the patient underwent a structured chemotherapy regimen, primarily involving the administration of TKIs, with a focus on imatinib. This therapeutic approach aligns with the evolving standards for managing GISTs and aims to optimize long-term outcomes.

The patient's follow-up included regular clinical assessments, imaging studies, and laboratory evaluations to monitor treatment response and detect potential recurrence. This comprehensive follow-up strategy underscores the commitment to ensuring the patient's well-being and the importance of a vigilant and proactive approach in the postoperative phase. The amalgamation of surgical expertise, adjuvant chemotherapy, and diligent follow-up collectively contributes to the holistic care and ongoing management of the patient.

## Discussion

Gastrointestinal stromal tumors are uncommon neoplasms characterized by their origin in the gastrointestinal tract and expression of CD117. The case presented here is emblematic of the diverse clinical and pathological characteristics that make GISTs a subject of intense study and clinical interest. Gastrointestinal stromal tumors exhibit variable clinical presentations, with some patients remaining asymptomatic. In contrast, others experience various symptoms, including abdominal pain, bleeding, obstruction, or a palpable abdominal mass [8,9]. The case report highlights the patient's prolonged one-year history of a progressively enlarging abdominal lump, abdominal distension, and associated symptoms of constipation and abdominal fullness.

The diagnostic evaluation of GISTs is multifaceted and relies on a combination of clinical, radiological, and histopathological findings. In this case, the USG of the abdomen and pelvis revealed a large, solid cystic lesion, while the CECT further delineated the mass's characteristics. These diagnostic modalities are pivotal in determining the size, location, and extent of GISTs. However, it is essential to note that the final diagnosis of GISTs typically hinges on histopathological analysis and immunohistochemistry, which can confirm the presence of CD117 and CD34 markers, aiding in the differentiation from other mesenchymal neoplasms [10].

As demonstrated in this case, surgical resection remains the primary curative modality for localized GISTs. The successful surgical management of this large exophytic GIST originating from the antral part of the stomach underscores the significance of accurate localization and complete resection. The case showcases the meticulous intraoperative approach to separating the cystic mass from the surrounding structures. Moreover, the importance of assessing the surgical margins through frozen sections cannot be overstated, as it aids in achieving negative margins, reducing the risk of recurrence [11].

The case also highlights the pivotal role of TKIs in managing GISTs. Imatinib mesylate (Gleevec), a TKI, has revolutionized the treatment of GISTs. In cases where complete resection is not feasible, neoadjuvant or adjuvant imatinib therapy can significantly improve disease-free survival and, in some instances, render tumors amenable to surgery [12,13]. Incorporating TKIs into the treatment strategy has improved the overall prognosis for patients with GISTs.

The case emphasizes the significance of a multidisciplinary approach in GIST management. Gastrointestinal stromal tumors are complex tumors requiring collaboration between surgeons, oncologists, pathologists, and radiologists. This approach ensures that each patient receives individualized treatment tailored to the specific characteristics of their GIST, whether through surgery, TKIs, or a combination of both [14].

The tumor size represents a critical parameter in GIST management, influencing both diagnostic considerations and treatment strategies. In this case, the tumor's substantial dimensions, as outlined in the diagnostic imaging section, played a pivotal role in determining the surgical approach and subsequent interventions. Patient survival, a paramount aspect of GISTs, is intricately linked to various factors, including tumor size, histopathological characteristics, and the efficacy of therapeutic interventions. While the specific details of patient survival, in this case, are not explicitly discussed in the current report, it underscores the broader point that effective GIST management requires a nuanced understanding of tumor characteristics, patient-specific factors, and the dynamic landscape of treatment options. Despite the strides made in GIST management, challenges persist, particularly in cases where the unpredictable behavior of GISTs and the emergence of resistance to TKIs pose clinical hurdles [15]. Consequently, ongoing research initiatives are crucial for exploring novel therapeutic options and refining our understanding of patient survival factors. The field of GIST management continues to evolve, driven by a synergy of clinical experience and emerging research findings, with the ultimate goal of optimizing patient outcomes.

## Conclusions

The case report of a giant exophytic GIST originating from the antral part of the stomach exemplifies the intricate clinical and pathological features of GISTs and the importance of a multidisciplinary approach to their management. Surgical resection, aided by TKIs when indicated, continues to be the cornerstone of GIST treatment. This case underscores the need for precise diagnosis, individualized treatment, and ongoing research to improve outcomes for patients with GISTs. In an era of evolving therapeutic options, GIST management remains a compelling study area, promising improved patient care and outcomes.
